# Bedaquiline micro-heteroresistance after tuberculosis treatment cessation.

**DOI:** 10.1056/NEJMc1815121

**Published:** 2019-05-30

**Authors:** Margaretha de Vos, Serej D. Ley, Helen Cox, Others

**Affiliations:** 1 DST-NRF Centre of Excellence for Biomedical Tuberculosis Research/South African Medical Research Council Centre for Tuberculosis Research, Division of Molecular Biology and Human Genetics, Faculty of Medicine and Health Sciences, Stellenbosch University, South Africa; 2 Institute of Infectious Disease and Molecular Medicine and Division of Medical Microbiology, Department of Pathology, Faculty of Health Sciences, University of Cape Town, South Africa

**Figure 1:**
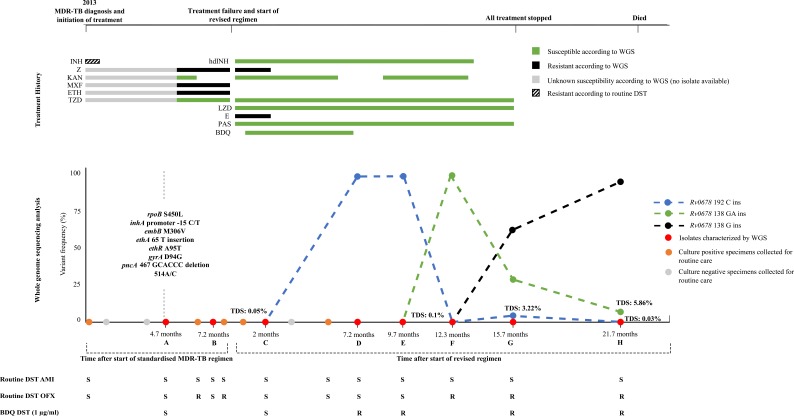
Chronology of the diagnosis and treatment of the case study Summary of treatment provision, genotypic drug resistance (based on whole genome sequencing, WGS), phenotypic bedaquiline drug susceptibility testing (DST, MGIT), targeted deep sequencing and treatment monitoring during standardised treatment and a subsequent individualised bedaquilinecontaining regimen. Overall, eight isolates (A-H) collected 4.7 months after initiation of standard treatment regimen until 6 months after all TB treatment was stopped underwent WGS, targeted deep sequencing of *Rv0678* and phenotypic bedaquiline DST. The patient was initially diagnosed with MDRTB with low-level isoniazid resistance using Genotype MTBDR*plus*, and treated with a standardised MDR-TB treatment regimen but remained culture positive. As per guidelines, subsequent isolates were phenotypically characterized for ofloxacin and amikacin susceptibility. Ofloxacin resistance was first noted 6 months after treatment initiation. All isolates remained susceptible to second-line injectables. At 8.1 months a revised regimen was initiated with the subsequent addition of bedaquiline (22 days after initiation of revised regimen) and withdrawal of pyrazinamide and ethambutol (2 months after initiation of revised regimen). Bedaquiline was administered for 6 months. The patient refused kanamycin at month 6 of the revised regimen for a duration of 2.4 months. The individualized regimen was continued until the outcome of treatment failure at 15 months. Phenotypic DST showed that all isolates with a variant frequency of >1% in *Rv0678* were resistant to bedaquiline at 1µg/ml in MGIT. Abbreviations: MDR-TB=multi-drug resistant tuberculosis; INH=isoniazid; Z=pyrazinamide; KAN=kanamycin; MXF=moxifloxacin; ETH=ethionamide; TZD=terizidone; hdIND=high dose isoniazid; KAN=kanamycin; LZD=linezolid; E=ethambutol; PAS=para-aminosalicyclic acid; BDQ=bedaquiline; WGS=whole genome sequencing; DST=drug susceptibility testing; ins=insertion; R=resistant; S=susceptible

Bedaquiline improves survival among individuals with multidrug-resistant tuberculosis (MDR-TB).^1^ We report a 65-year old HIV-negative South African male diagnosed in 2013 with MDR-TB (resistant to rifampicin and isoniazid; phenotypically susceptible to a fluoroquinolone and amikacin). Baseline X-ray showed bilateral TB disease with left apex cavitation. He initiated standardised treatment including moxifloxacin, pyrazinamide, kanamycin, ethionamide, isoniazid and terizidone. After initial sputum culture conversion (month 3) and clinical improvement, the patient reconverted to culture positive and developed bilateral cavitation. Following detection of phenotypic ofloxacin resistance (month 6), treatment was revised (month 8) to include high-dose isoniazid, ethambutol, pyrazinamide, terizidone, linezolid, para-aminosalicylic acid and kanamycin (Figure 1). Bedaquiline was added 22 days later and administered for 6 months.^2^ The patient remained culture positive (treatment failure) and treatment was stopped 15 months after revision of the regimen. The patient died 7 months later.

Overall, eight *M. tuberculosis* isolates (A-H) underwent whole genome sequencing (WGS), targeted deep sequencing^3^ of *Rv0678* and phenotypic bedaquiline resistance testing. WGS of isolate A collected 4.7 months after standard MDR-TB treatment initiation revealed a Beijing strain with mutations conferring resistance to rifampicin, isoniazid, ethambutol, ethionamide, fluoroquinolones, pyrazinamide and streptomycin (Figure 1). WGS of isolate C, collected 2 months after treatment revision, suggested that bedaquiline (to which the isolate was phenotypically susceptible) was added to a regimen with 5 potentially effective drugs. Targeted deep sequencing of isolate C showed a base pair insertion in *Rv0678**^4^* at a variant frequency of 0.05% (position 192), which was not present in isolate B taken before bedaquiline treatment. Isolate D, collected after bedaquiline cessation, showed the presence of this insertion in >90% of the bacterial population. The frequency of the *Rv0678* 192 insertion decreased in subsequent isolates, but two different insertions in *Rv0678* emerged (GA and G at position 138, isolates F and G, respectively). The G insertion at position 138 became fixed after all treatment was stopped (isolates G and H). Isolates D, E, F, G, and H were phenotypically resistant to bedaquiline.

This case demonstrates the emergence of bedaquiline resistance despite the presence of five potentially effective drugs and good adherence (based on clinical notes). The emergence of *Rv0678* variants, after completion of 6 months of bedaquiline treatment, demonstrates the risk of resistance amplification after cessation of a drug with a long half-life (5.5 months for bedaquiline).^5^
