# Supplementary material for: Bedaquiline micro-heteroresistance after tuberculosis treatment cessation
Source: N Engl J Med. Author manuscript; Available in PMC 2019 May 30. (PMC6518951; doi:10.1056/NEJMc1815121)
Supplement: Supplementary file 1 [file NEJM-2019-1815121-s1.pdf]

Content **Page**

Methods: ………………………………………………………………………………………..... 2
Results and Discussion: ……………………………………………………………………... 3
Acknowledgements: ………………………………………………………………………… 5
Disclaimer: ……………………………………………………………………………………... 6
References: ……………………………………………………………………………………...6
Supplementary tables: …………………………………………………………………….. 8
Table S1: ………………………………………………………………………………………8
Table S2: …………………………………………………………………………………… 9
Table S3: …………………………………………………………………………………...10
Supplementary figures: …………………………………………………………………….. 11
Figure S1: ………………………………………………………………………………. 12

SUPPLEMENTAL APPENDIX:

Methods:

Whole genome sequencing (WGS) of serial patient isolates

Stored clinical isolates spanning most of the course of treatment and several months after cessation of treatment were re-cultured and DNA was extracted as previously described.[1] Eight isolates were available for WGS using the Illumina NextSeq platform at a median coverage of 62x. Sequencing reads were mapped to a reference genome (*Mycobacterium tuberculosis,* H37Rv, Genbank: AL123456) with variant calling and annotation performed as previously described.[2] All identified variants were visually inspected using Tablet [3]. In addition, TB profiler [4] was used to ascertain the genotypic drug susceptibility (DST) profile. Raw sequencing reads have been deposited at the European Nucleotide Archive (project accession nr: PRJEB32109). All variants identified in *Rv0678* were confirmed by Sanger sequencing using the following primer pair (forward primer: 5'agagttccaatcatcgccct 3'; reverse primer: 5'tgctcatcagtcgtcctctc 3').

Targeted deep sequencing and data analysis

Targeted deep sequencing was done as previously described[5] using the following primer pair; forward primer: 5'ACCCAACTGAATGGAGCGAAACTTGTGAGCGTCAACGAC G 3'; reverse primer: 5' ACGCACTTGACTTGTCTTCGGTTGCTCATCAGTCGTCCT 3'. Raw sequencing reads have been deposited at Bioproject at NCBI (project accession nr: PRJNA531707). Data analysis was done using the Allele-specific alignment pipeline (ASAP)[5] using two aligners, Bowtie[6] and BWA[7]. Only short insertions and deletions identified in both alignment files generated by BWA and Bowtie were included for further analysis.

Bedaquiline phenotypic drug susceptibility testing

Phenotypic DST for bedaquiline was conducted using the BACTEC MGIT 960 system and EpiCentre software equipped with the TB eXist module for DST.[8] Briefly, each *M. tuberculosis* isolates was subcultured in MGIT supplemented with Oleic Albumin Dextrose Catalase (OADC) until a positive growth was observed. Thereafter 0.5 mL of each subculture was added to respective MGIT tubes supplemented with 0.8 mL of OADC and containing a final bedaquiline concentration of 1µg/ml. Concurrently, a growth control was prepared by inoculating 0.5 mL of a 1:100 dilution of each subculture into respective MGIT tubes supplemented with 0.8 mL of OADC without bedaquiline. The laboratory strain, H37Rv (ATCC 27294), was used as a susceptible control and the *M. tuberculosis* strain (BCCM/ITM 121749), obtained from Belgium co-ordinated collection of micro-organisms (A63P mutation in *atpE*) was used as a resistant control. Isolates were considered resistant if the growth index of the bedaquiline containing tube was greater than 100 when the growth control reached a growth index of 400.

Results and discussion:

A 65-year old male was diagnosed in 2013 in South Africa with MDR-TB using Xpert MTB/RIF and Genotype MTBDR*plus*. Baseline chest x-ray showed right hilar infiltrate, consolidation in the right apex and cavitation in the left apex. He initiated a standardised regimen including moxifloxacin, pyrazinamide, kanamycin, ethionamide, isoniazid and terizidone, as per national guidelines within two days of diagnosis. Isolates taken at diagnosis and initiation of treatment were culture-positive and susceptible to ofloxacin and amikacin based on routine phenotypic DST (National Health Laboratory Services, Green Point, South Africa). Isoniazid was withdrawn 28 days after treatment initiation. Follow-up routine sputum specimens taken 42 days and 3.4 months after treatment initiation were acid-fast bacilli (AFB) smear and culture negative (Table S1). Four subsequent sputum specimens collected four to eight months after treatment initiation were smear- and culture-positive. Kanamycin was stopped six months after treatment initiation. The sputum specimen taken six months after initiation of standard treatment showed phenotypic resistance to ofloxacin using phenotypic DST and the patient was classified as failure of treatment. Month six chest x-ray showed extensive fibrosis in the left lung and cavitation in both apices. At month eight, treatment was revised to include high-dose isoniazid (800 mg), ethambutol, pyrazinamide, terizidone, linezolid, para-aminosalicylic acid (PAS) and kanamycin. Bedaquiline was added 22 days later and administered for six months per the South African Bedaquiline Clinical Access Program.[9] The patient was admitted at a TB inpatient facility for the first two months of bedaquiline treatment. By examination of the patient's treatment card and patient interview, adherence to bedaquiline during both in-patient and out-patient treatment was subjectively assessed as good, although strict direct observation of treatment was not practised. Pyrazinamide and ethambutol were stopped at 2.3 months following revised regimen initiation due to persistent arthralgia and changes in vision. The patient refused kanamycin at month six for a duration of 2.4 months after more than twelve months of injectable treatment. Kanamycin and high-dose isoniazid were stopped at 13 months (isoniazid due to vision problems). The physician decided to stop all treatment at 15.7 months after which the patient was transitioned to palliative care and died seven months later. All sputum cultures subsequent to stopping of all treatment were positive.

During the course of treatment a total of 19 sputum cultures were requested (Table S1), of which ten could be retrieved from the National Health Laboratory Service (NHLS) in Cape Town. From the ten isolates retrieved, eight were re-cultured for next generation sequencing (NGS), while two isolates lost viability. All eight isolates differed by a maximum of five variants, implying *in vivo* evolution rather than reinfection with different strains. WGS of the first available isolate (isolate A) taken 4.7 months after initiation of the standard MDR-TB treatment regimen showed that the patient was infected with a Beijing strain which harboured mutations in *rpoB* (S450L), *inhA* promoter region (-15 C/T), *embB* (M306V), *ethA* (65 T insertion), *ethR* (A95T), *gyrA* (D94G), *pncA* (467 GCACCC deletion) and *rrs* (514A/C) associated with resistance to rifampicin, isoniazid, ethambutol, ethionamide, fluoroquinolones, pyrazinamide and streptomycin, respectively. All of these resistance-causing

mutations were present in 100% of the sequencing reads. The detection of a D94G substitution in *gyrA*, which confers resistance to fluoroquinolones, suggests that the classification of fluoroquinolone susceptibility by phenotypic DST performed at the routine laboratory on the same isolate was incorrect. The isolate taken 7.2 months after treatment initiation (isolate B) did not show amplification of resistance, but showed the loss of a variant in *rpoC* (A734G) (Table S2, Figure S1). According to the WGS data, the patient received only two potentially effective drugs (kanamycin and terizidone) (Figure 1). Phenotypic resistance to ofloxacin was first detected by the routine laboratory on the specimen collected 6 months after treatment initiation. Targeted deep sequencing (TDS) of isolate B did not detect any underlying variants in *Rv0678*, the gene associated with resistance to bedaquiline.[10]

WGS analysis of isolate C collected 2 months after initiation of the individualized pre-XDR treatment regimen and 10 weeks after the initiation of bedaquiline, showed the presence of wild-type sequences for the genes associated with resistance to second-line injectables (*rrs* (1401 region), PAS (*thyA*, *folC*, *dfrA*, *ribD*), linezolid (*rrl*, *rplC),* terizidone (*ddl, cycA, alr, ald)* and bedaquiline *(Rv0678)* suggesting that the patient likely received five effective anti-TB drugs (high dose isoniazid, kanamycin, linezolid, terizidone and PAS) at the time of addition of bedaquiline. TDS however showed the presence of micro-heteroresistance against bedaquiline with a C insertion at position 192 in *Rv0678* in 0.05% of the reads (Table S3) despite phenotypic susceptibility to bedaquiline and genotypic susceptibility on WGS. In the isolate collected one week after bedaquiline treatment was stopped (isolate D), WGS and TDS showed that the *Rv0678* 192 variant was fixed and TDS showed the presence of multiple low frequency (>0.1%) indels (insertions and deletions) in codons 194 to 198 in *Rv0678*. In addition, a fixed variant in *Rv2839c* (S347P) was also observed by WGS (Table S2). Isolate D was also phenotypically resistant to bedaquiline. Subsequently, the 192 C insertion decreased to 0.1% of reads in isolate F (taken 12.3 months after the start of the individualized regimen and five months after the cessation of bedaquiline) and was replaced with a different *Rv0678* variant (GA insertion at gene position 138) and a second *rpoB* variant (S582A) according to WGS. In the subsequent isolates taken 15.7 months (isolate G) and 21.7 months (isolate H) after initiation of the revised regimen, WGS and TDS showed the systematic decrease of the *Rv0678* 138 GA insertion over time and the gain of a third *Rv0678* variant (G insertion at position 138). WGS showed the emergence of an *rpoC* (V483G) variant in this isolate. These *Rv0678* 138 G insertion and *rpoC* V483G were found with a variant frequency of 96% in the last isolate taken. The systematic gain and loss of *Rv0678* variants identified by WGS was confirmed by Sanger sequencing (data not shown). All isolates with a variant frequency of >1% in *Rv0678* were resistant to bedaquiline at a concentration of 1 µg/ml in MGIT media.

The identification of a subpopulation of bacilli harbouring a variant in *Rv0678* 10 weeks after addition of bedaquiline suggest that bedaquiline resistance emerged soon after its inclusion in the revised MDR-TB regimen and was subsequently selected as treatment continued. Following the withdrawal of bedaquiline a further gain and loss of *Rv0678* variants were observed over the course of the long half-

life of bedaquiline (5.5 months) [11]. These results suggest that new *Rv0678* variants can emerge while plasma concentration of bedaquiline are decreasing. Alternatively, the variants could have emerged in different lesions prior to the withdrawal of bedaquiline and subsequently observed as these lesions ruptured into the airways. Our data also suggests that bedaquiline resistance in this patient developed despite treatment with a background regimen containing five anti-TB drugs that were likely effective based on susceptibility. This highlights our lack of comprehensive understanding of resistance emergence during treatment. No amplification of mutations conferring resistance to the five companion drugs were identified in the serial patient isolates, suggesting that the isolates would still be susceptible to those drugs (high dose isoniazid, kanamycin, linezolid, terizidone and PAS). The presence of bedaquiline-resistant *M. tuberculosis* following cessation of bedaquiline and cassation of all TB treatment because of treatment failure poses a potential transmission risk and threatens the longevity of this new drug. Monitoring of pre-existing and emerging bedaquiline resistance should be a priority among patients with delayed sputum culture conversion and those with positive sputum cultures post bedaquiline cessation.

Four of the nine variable loci were in *rpoB* and *rpoC* genes, associated with rifampicin resistance or fitness compensatory mechanisms. Secondary *rpoB* mutations have been shown to improve growth characteristics and fitness rates in a BCG model[12]. Numerous studies have investigated the role of *rpoC* mutations in compensating for the loss of fitness due to *rpoB* mutations [13-16]. Three of the four variants were however transient and only one variant (V483G) was fixed in the last available isolate. We have reported the V483G substitution to be the most frequent *rpoC* variant in our setting, which is also associated with transmission clusters as defined by IS*6110* fingerprinting [14].

In summary, this case demonstrates the rapid acquisition of bedaquiline resistance in the presence of five likely effective drugs. There was no evidence of poor adherence to treatment over this time. The emergence of *Rv0678* variants, after completion of six months bedaquiline, demonstrates the risk of resistance amplification after cessation of a drug with a long half-life (5.5 months for bedaquiline)[11]. These data highlight the potential utility of sequencing approaches to guide treatment and monitor resistance emergence and the need to incorporate new drugs into more effective regimens from the start of treatment.

Acknowledgements

This work is supported by the National Research Foundation, the South African Medical Research Council (SA MRC) and the Stellenbosch University Faculty of Medicine Health Sciences. The content is solely the responsibility of the authors and does not necessarily represent the official views of the SA MRC. The work is supported by National Institutes of Health (NIH) grant R01AI131939 (DM Engelthaler and JZ Metcalfe). The content is solely the responsibility of the authors and does not necessarily represent the official views of the NIH. HC acknowledges support from a Wellcome Trust fellowship (ref 099818/Z/12/Z). MdV, AvR and SL are supported by TORCH funding through the

Flemish Fund for Scientific Research (FWO G0F8316N). SL has also been supported by the Swiss National Science Foundation (P2BSP3_165379). The sequencing work was performed at the Translational Genomics Research Institute and supported by the Bill & Melinda Gates Foundation Grant OPP1115887 (M Schito) for the ReSeqTB sequencing platform. GT acknowledges support from the EDCTP2 programme supported by the European Union (grant number SF1401 – OPTIMAL DIAGNOSIS).

Disclaimer

Use of trade names is for identification only and does not constitute endorsement by the U.S. Department of Health and Human Services, the U.S. Public Health Service, or the Centers for Disease Control and Prevention. The findings and conclusions in this report are those of the authors and do not necessarily represent the views of the Centers for Disease Control and Prevention.

References:

1. Warren R, de Kock M, Engelke E, et al. Safe Mycobacterium tuberculosis DNA extraction method that does not compromise integrity. Journal of clinical microbiology 2006;44:254-6.
2. Black PA, de Vos M, Louw GE, et al. Whole genome sequencing reveals genomic heterogeneity and antibiotic purification in Mycobacterium tuberculosis isolates. BMC genomics 2015;16:857.
3. Milne I, Bayer M, Cardle L, et al. Tablet--next generation sequence assembly visualization. Bioinformatics 2010;26:401-2.
4. Coll F, McNerney R, Preston MD, et al. Rapid determination of anti-tuberculosis drug resistance from whole-genome sequences. Genome Med 2015;7:51.
5. Colman RE, Anderson J, Lemmer D, et al. Rapid Drug Susceptibility Testing of Drug-Resistant Mycobacterium tuberculosis Isolates Directly from Clinical Samples by Use of Amplicon Sequencing: a Proof-of-Concept Study. Journal of clinical microbiology 2016;54:2058-67.
6. Langmead B. Aligning short sequencing reads with Bowtie. Curr Protoc Bioinformatics 2010;Chapter 11:Unit 11 7.
7. Li H, Durbin R. Fast and accurate short read alignment with Burrows-Wheeler transform. Bioinformatics 2009;25:1754-60.
8. Springer B, Lucke K, Calligaris-Maibach R, Ritter C, Bottger EC. Quantitative drug susceptibility testing of Mycobacterium tuberculosis by use of MGIT 960 and EpiCenter instrumentation. Journal of clinical microbiology 2009;47:1773-80.
9. Conradie F, Meintjes G, Hughes J, et al. Clinical access to Bedaquiline Programme for the treatment of drug-resistant tuberculosis. S Afr Med J 2014;104:164-6.
10. Andries K, Villellas C, Coeck N, et al. Acquired resistance of Mycobacterium tuberculosis to bedaquiline. PloS one 2014;9:e102135.

11. McLeay SC, Vis P, van Heeswijk RP, Green B. Population pharmacokinetics of bedaquiline (TMC207), a novel antituberculosis drug. Antimicrobial agents and chemotherapy 2014;58:5315-24.

12. Meftahi N, Namouchi A, Mhenni B, Brandis G, Hughes D, Mardassi H. Evidence for the critical role of a secondary site rpoB mutation in the compensatory evolution and successful transmission of an MDR tuberculosis outbreak strain. J Antimicrob Chemother 2016;71:324-32.

13. Comas I, Borrell S, Roetzer A, et al. Whole-genome sequencing of rifampicin-resistant Mycobacterium tuberculosis strains identifies compensatory mutations in RNA polymerase genes. Nature genetics 2012;44:106-10.

14. de Vos M, Muller B, Borrell S, et al. Putative compensatory mutations in the rpoC gene of rifampin-resistant Mycobacterium tuberculosis are associated with ongoing transmission. Antimicrobial agents and chemotherapy 2013;57:827-32.

15. Brandis G, Hughes D. Genetic characterization of compensatory evolution in strains carrying rpoB Ser531Leu, the rifampicin resistance mutation most frequently found in clinical isolates. J Antimicrob Chemother 2013;68:2493-7.

16. Brandis G, Wrande M, Liljas L, Hughes D. Fitness-compensatory mutations in rifampicin-resistant RNA polymerase. Mol Microbiol 2012;85:142-51.

Supplementary tables

Table S1: *M. tuberculosis* isolates collected over the course of treatment

Isolate taken		Smear microscopy	Culture	GenoType MTBDR*plus*		Phenotypic DST		WGS isolate label
				**Rifampicin**	**Isoniazid**	**Amikacin**	**Ofloxacin**	
**Time after initiation of treatment**	0 days*	Neg	Pos	R	R	S	S	
	2 days	2+	Pos	R	R	S	S	
	41 days	Neg	Neg					
	3.4 months	Neg	Neg					
	4.7 months	Scanty	Pos	R	R	S	S	**A**
	6.2 months	3+	Pos	R	R	S	R	
	7.2 months	Scanty	Pos	R	R	S	S	**B**
	8 months	3+	Pos	R	R	S	R	
**Time after initiation of revised MDR regimen**	20 days	3+	Pos	R	R	S	S	
	22 days	3+	Pos	R	R	S	S	
	2 months	Scanty	Pos	R	R	S	S	**C**
	3.5 months	Neg	Neg					
	5.6 months	Neg	Pos	ND	ND			
	7.2 months	Neg	Pos	R	R	S	S	**D**
	9.7 months	Scanty	Pos	R	R	S	R	**E**
	10.3 months	Neg	NTM					
	12.3 months	Neg	Pos	R	R	S	R	**F**
	15.7 months	Neg	Pos	R	R	S	R	**G**
	21.7 months	3+	Pos	R	R	S	R	**H**

Definitions for abbreviations: Tx = treatment; MTB = *M. tuberculosis*; WGS = whole genome sequencing; Pos = positive; Neg = negative; R = resistant; S = sensitive; ND = not done; NTM = non-tuberculosis mycobacteria

* Xpert MTB/RIF MTB complex positive and rifampicin resistant

Table S2: Variants identified through whole genome sequencing in serial isolates cultured from the patient during treatment.

Isolate	Time relative to the initiation of the revised regimen	Variant frequency of variants identified by whole genome sequencing*								
		rpoC A734G	*rpoC* L823M	*Rv0678* 192 G ins	*Rv2839c* S347P	*rpoB* S582A	*Rv0678* 138 GA ins	*Rv0678* 138 G ins	*rpoC* V483G	*Rv3777* 147 Syn
**A**	**-102 days**	86 (14/85)	0	0	0	0	0	0	0	0
**B**	**-29 days**	0	0	0	0	0	0	0	0	0
**C**	**62 days**	0	100 (36/36)	0	0	0	0	0	0	0
**D**	**7.2 months**	0	0	100 (56/56)	100 (56/56)	0	0	0	0	0
**E**	**9.7 months**	0	0	100 (56/56)	100 (56/56)	0	0	0	0	0
**F**	**12.3 months**	0	0	0	0	100 (81/81)	100 (75/75)	0	0	0
**G**	**15.7 months**	0	0	0	0	35 (24/67)	25 (18/71)	63 (45/71)	65 (52/79)	52 (36/68)
**H**	**21.7 months**	0	0	0	0	0	0	96 (79/82)	95 (65/68)	91 (73/80)

* In brackets - Number of reads with variant/total number of reads

Table S3: Minority populations identified through targeted deep sequencing of *Rv0678*

		138 G insertion		138 GA insertion		139 T insertion		192 G insertion		193 G deletion	
**Isolate**	**Date#**	**WGS***	**TDS***	**WGS***	**TDS***	**WGS***	**TDS***	**WGS***	**TDS***	**WGS***	**TDS***
**A**	**-102 days**	0	-	0	-	0	-	0	-	0	-
**B**	**-29 days**	0	0	0	0	0	0	0	0	0	0
**C**	**62 days**	0	0	0	0	0	0	0	0.05 (7/15886)	0	0
**D$**	**7.2 months**	0	0	0	0	0	0	100 (56/56)	96.66 (17551/18158)	0	0
**E**	**9.7 months**	0	-	0	-	0	-	100 (56/56)	-	0	-
**F**	**12.3 months**	0	0	100 (75/75)	97.52 (13299/13638)	0	0	0	0.1 (13/13638)	0.37 (50/13638)	0
**G**	**15.7 months**	63 (45/71)	65.48 (9317/14230)	25 (18/71)	28.35 (4034/14230)	0	0	0	3.22 (461/14230)	0.28 (39/14230)	0
**H**	**21.7 months**	96 (79/82)	91.68 (13029/14212)	0	5.86 (832/14212)	0	0.14 (19/14212)	0	0.03 (4/14212)	0	0

Definitions of abbreviations: ins = insertion; WGS = whole genome sequencing; TDS = targeted deep sequencing; "-" = not done

# relative to initiation of revised MDR-TB regimen

* In brackets - Number of reads with variant/total number of reads

$ other low frequency variants identified in isolate D: 194 T insertion (0.32%), 195 C insertion (0.22%); 196 T insertion (0.22%), 197 T insertion (0.18%), 198 T insertion (0.17%)

Supplementary figures

Legend to Figure S1: Chronology of the diagnosis and treatment of the case

Summary of treatment provision, genotypic drug resistance (based on whole genome sequencing, WGS), phenotypic bedaquiline drug susceptibility testing (DST, MGIT), targeted deep sequencing and treatment monitoring during standardised treatment and a subsequent individualised bedaquiline-containing regimen. Overall, eight isolates (A-H) collected 4.7 months after initiation of standard treatment regimen until 6 months after all TB treatment was stopped underwent WGS, targeted deep sequencing of *Rv0678* and phenotypic bedaquiline DST. The patient was initially diagnosed with MDR-TB with low-level isoniazid resistance using Genotype MTBDR*plus*, and treated with a standardised MDR-TB treatment regimen but remained culture positive. Phenotypic As per guidelines, subsequent isolates were phenotypically characterized for ofloxacin and amikacin susceptibility. Ofloxacin resistance was first noted 6 months after treatment initiation. All isolates remained susceptible to second-line injectables. At 8.1 months a revised regimen was initiated with the subsequent addition of bedaquiline (22 days after initiation of revised regimen) and withdrawal of pyrazinamide and ethambutol (2 months after initiation of revised regimen). Bedaquiline was administered for 6 months. The patient refused kanamycin at month 6 of the revised regimen for a duration of 2.4 months. The individualized regimen was continued until the outcome of treatment failure at 15 months. Phenotypic DST showed that all isolates with a variant frequency of >1% in *Rv0678* were resistant to bedaquiline at 1µg/ml in MGIT.

Abbreviations: MDR-TB=multi-drug resistant tuberculosis; INH=isoniazid; Z=pyrazinamide; KAN=kanamycin; MXF=moxifloxacin; ETH=ethionamide; TZD=terizidone; hdIND=high dose isoniazid; KAN=kanamycin; LZD=linezolid; E=ethambutol; PAS=para-aminosalicyclic acid; BDQ=bedaquiline; WGS=whole genome sequencing; DST=drug susceptibility testing; ins=insertion; R=resistant; S=susceptible

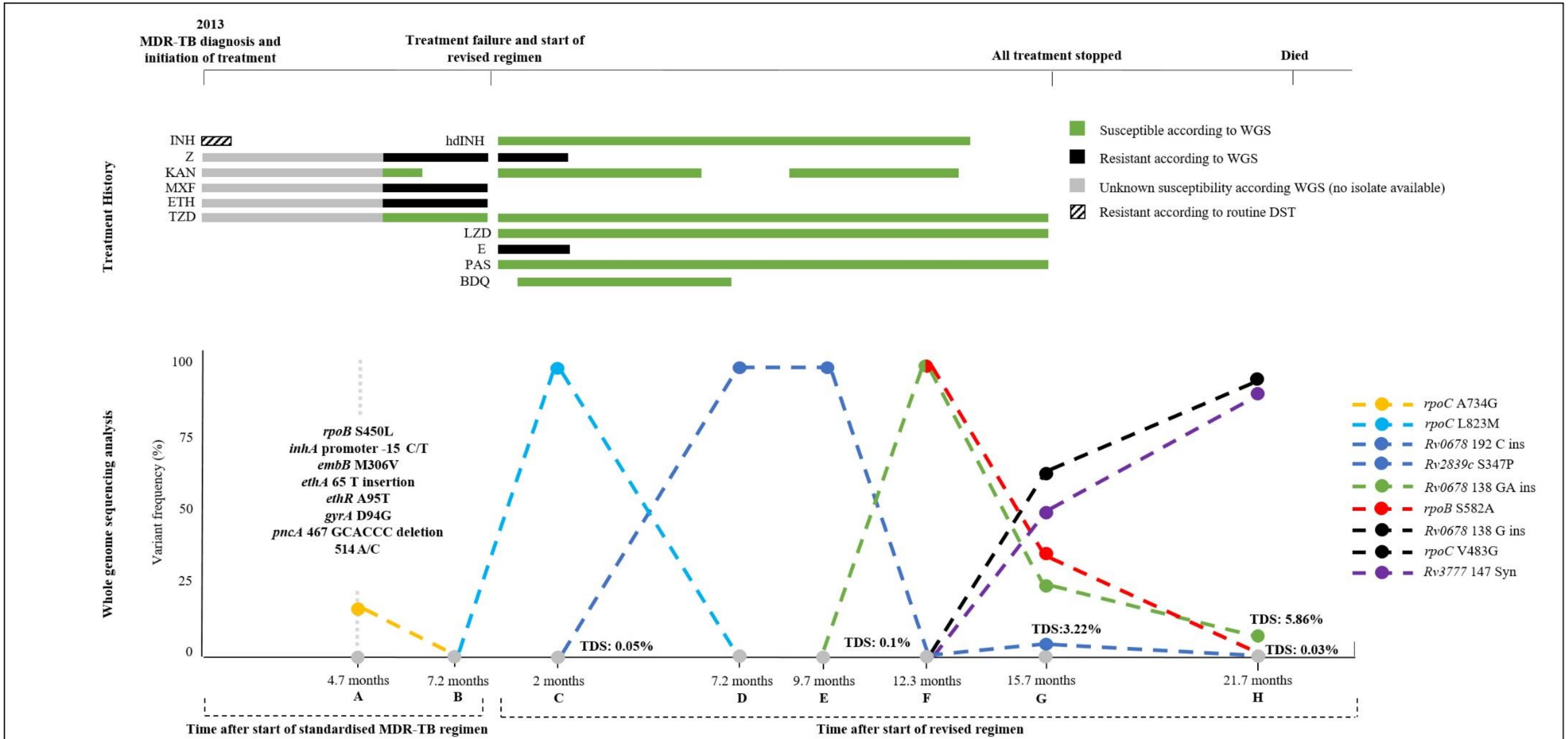

	A	B	C	D	E	F	G	H
Routine DST AMI	S	S	S	S	S	S	S	S
Routine DST OFX	S	S	S	S	R	R	R	R
BDQ DST (1 µg/ml)	S		S	R	R		R	R
